# Fibrogenic Potential of Human Multipotent Mesenchymal Stromal Cells in Injured Liver

**DOI:** 10.1371/journal.pone.0006657

**Published:** 2009-08-17

**Authors:** Reto M. Baertschiger, Véronique Serre-Beinier, Philippe Morel, Domenico Bosco, Marion Peyrou, Sophie Clément, Antonino Sgroi, André Kaelin, Leo H. Buhler, Carmen Gonelle-Gispert

**Affiliations:** 1 Surgical Research Unit, Department of Surgery, University Hospital Geneva, Geneva, Switzerland; 2 Cell Isolation and Transplantation Center, Department of Surgery, University Hospital Geneva, Geneva, Switzerland; 3 Department of Pathology and Immunology, Medical School of Geneva, Geneva, Switzerland; 4 Department of Pediatric Orthopedics, Children's Hospital, University Hospital of Geneva, Geneva, Switzerland; Tufts University, United States of America

## Abstract

Multipotent mesenchymal stromal cells (MSC) are currently investigated clinically as cellular therapy for a variety of diseases. Differentiation of MSC toward endodermal lineages, including hepatocytes and their therapeutic effect on fibrosis has been described but remains controversial. Recent evidence attributed a fibrotic potential to MSC. As differentiation potential might be dependent of donor age, we studied MSC derived from adult and pediatric human bone marrow and their potential to differentiate into hepatocytes or myofibroblasts *in vitro* and *in vivo*. Following characterization, expanded adult and pediatric MSC were co-cultured with a human hepatoma cell line, Huh-7, in a hepatogenic differentiation medium containing Hepatocyte growth factor, Fibroblast growth factor 4 and oncostatin M. *In vivo*, MSC were transplanted into spleen or liver of NOD/SCID mice undergoing partial hepatectomy and retrorsine treatment. Expression of mesenchymal and hepatic markers was analyzed by RT-PCR, Western blot and immunohistochemistry. *In vitro*, adult and pediatric MSC expressed characteristic surface antigens of MSC. Expansion capacity of pediatric MSC was significantly higher when compared to adult MSC. In co-culture with Huh-7 cells in hepatogenic differentiation medium, albumin expression was more frequently detected in pediatric MSC (5/8 experiments) when compared to adult MSC (2/10 experiments). However, in such condition pediatric MSC expressed alpha smooth muscle more strongly than adult MSC. Stable engraftment in the liver was not achieved after intrasplenic injection of pediatric or adult MSC. After intrahepatic injection, MSC permanently remained in liver tissue, kept a mesenchymal morphology and expressed vimentin and alpha smooth muscle actin, but no hepatic markers. Further, MSC localization merges with collagen deposition in transplanted liver and no difference was observed using adult or pediatric MSC. In conclusion, when transplanted into an injured or regenerating liver, MSC differentiated into myofibroblasts with development of fibrous tissue, regardless of donor age. These results indicate that MSC in certain circumstances might be harmful due to their fibrogenic potential and this should be considered before potential use of MSC for cell therapy.

## Introduction

Currently, orthotopic liver transplantation is the only treatment for fulminant and end-stage liver diseases. As patients die on transplant waiting lists due to insufficient numbers of organ donors, alternative therapies need urgently to be developed. Adult and embryonic stem cells are potential options to overcome the lack of organ availability. Studies have shown that bone marrow cells migrate and integrate into the liver suggesting that the bone marrow contains hepatocyte progenitor cells [1]–[3]. One potential candidate is the multipotent mesenchymal stromal cells (MSC). These cells present criteria of a transplantable stem cell, especially for the use of autologous cell transplantation, as MSC are easily accessible and present high expansion potential *in vitro*.

 MSC have been identified in the bone marrow [4] and in various human tissues [5], [6]. MSC are able to differentiate into chondrocytes, adipocytes and osteoblasts 4, 7. Several studies indicate that MSC might have the ability to cross cell lineage boundaries and differentiate towards or into hepatocytes *in vitro*. A two-step protocol, using hepatocyte growth factor (HGF) and oncostatin M induced hepatocyte specific gene expression in clonal expanded MSC derived from bone marrow and human cord-blood [8]. Further, co-culturing rat MSC with isolated rat liver cells in medium containing several growth factors induced expression of hepatocyte-specific genes [9].

However, therapeutic potential of MSC for supporting liver regeneration is not clear. MSC transplantation into rodents with carbon tetrachloride (CCl4) -induced liver injury resulted in reduced liver fibrosis [10], [11]. In contrast, others showed that reduction of fibrosis was not influenced by transplantation of MSC [12]. In some mouse models of liver injury, using either partial hepatectomy or injection of toxic agents such as CCl4 or acetaminophen, MSC engrafted into the liver and albumin expression was detected [13], [14].

Further investigations are required to determine if MSC represent a therapeutic option for liver disease. Importance of donor age of MSC on engraftment and differentiation into hepatocytes has not been evaluated. Age is critical for chromosomal stability [15], telomerase activity [16], [17] and thus differentiation potential. In this study, we investigated the potential of MSC derived from human adult (aMSC) and pediatric bone marrow (pMSC) to differentiate into hepatocytes. When co-cultured with Huh-7 cells, a human hepatoma cell line, in medium containing endodermal growth factors, pMSC expressed albumin more frequently compared to aMSC. Independently of age, MSC transplanted into liver did not differentiate into hepatocytes but remained mesenchymal and expressed collagen and alpha smooth muscle actin (αSMA), a marker for myofibroblast differentiation. These results show that MSC exposed to an injured liver environment in mice can contribute to a fibrogenic tissue. As MSC are currently tested clinically to treat bone diseases [18], [19] or graft versus host disease [20], it is important to assess a potential harmful effect on liver tissue.

## Results

### Characterization of adult and pediatric bone marrow-derived multipotent mesenchymal stromal cells

**Figure 1:**
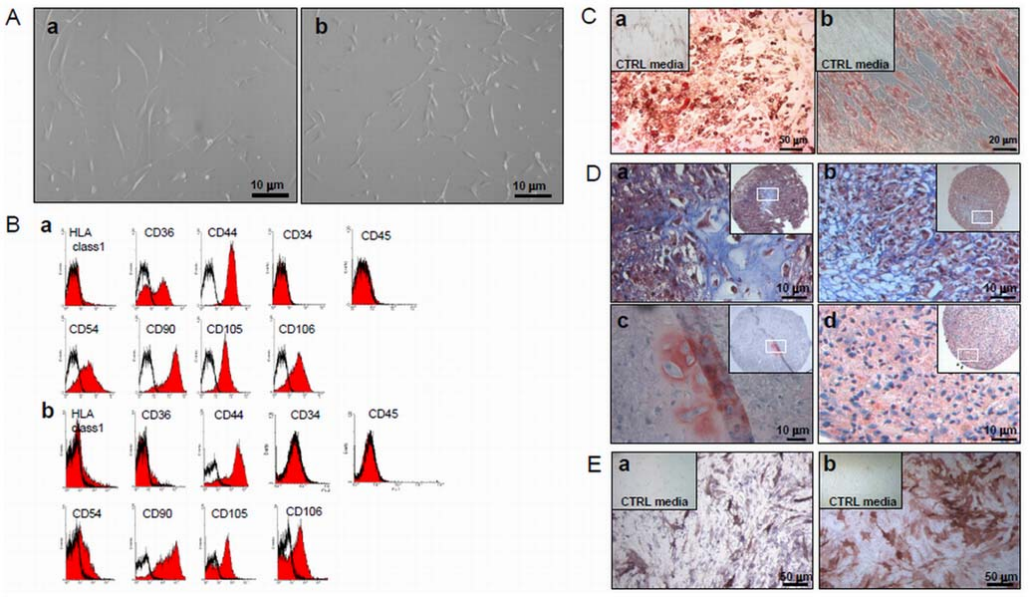
Characterization of adult and pediatric multipotent mesenchymal stromal cells. A) Fibroblast-like morphology of adult (a) and pediatric (b) MSC. Both cell types showed similar morphology. B) Flow cytometry of cells stained for CD34, CD36, CD44, CD45, CD54, CD90, CD105, CD106, HLA ABC and isotype controls showing similar expression of surface antigens in adult (a) and pediatric (b) MSC. A representative example of adult and pediatric MSC is shown. Red histograms: specific marker; unfilled black histograms: isotype controls. C) Adipogenic differentiation was induced with adipocyte differentiation medium. After 3 weeks aMSC (a) and pMSC (b) were fixed and stained with Oil-red-O. In control medium (Insets), very few cells contained lipid droplets. In adipogenic differentiation medium a majority of cells contained lipid droplets stained in red. D) Chondrogenic differentiation was induced in MSC pellet culture in chondrogenic differentiation medium. After 4 weeks, pellets were fixed and sections were analyzed using Masson trichrome staining (a,b blue) and anti-collagen type II immunohistochemistry (c,d red). The images represent zoomed portion (delimitated by white frames) of the picture shown in the insets. The pellet structure appeared compact and contained abundant collagen fibrils. E) Osteogenic differentiation was induced by culturing aMSC (a) and pMSC (b) in osteogenic differentiation medium. After 4 weeks, cells were washed and incubated with Fast red and Sodium alpha-naphtyl phosphate solutions for 30 min at 4°C. Nuclei were stained with hemalun. MSC expressing alkaline phosphatase showed brown colored cytoplasm and displayed typical star-shaped cell morphology. In control medium (Insets), only few cells showed alkaline phosphatase expression.

We isolated MSC from 38 adult donors (mean age: 59±12 years) and 11 pediatric donors (mean age: 10±3 years). Adult MSC were expanded for 5±3 passages to 19 population doublings (PD), whereas pediatric MSC (pMSC) were expanded for 8±4 passages to 24 PD. The difference of passage numbers and expansion potential between the 2 groups were statistically significant (p = 0.016 and p = 0.00006, respectively). Adult MSC and pMSC showed a similar fibroblast-like morphology (Fig. 1A). Expanded cells were characterized by FACS analysis and were negative for HLA class1, CD34 and CD45, but were positive for CD44, CD54, CD90 and CD105 (Fig. 1B). CD36 was not expressed in pMSC whereas a double population was observed in aMSC. The expression level of CD106 varied between donors (not shown). This expression pattern remained unchanged from passages 3 to 7. CD31 and CD133 were negative in both populations (data not shown).

### Adipogenic, chondrogenic and osteogenic differentiation of adult and pediatric MSC

Adult MSC and pMSC could be differentiated into adipocytes as shown by oil-red-O staining of lipid droplets (Fig. 1C), into chondrocytes as shown by Masson's trichrome and immunohistochemistry for collagen type II (Fig. 1D) and into osteoblasts expressing alkaline phosphatase activity (Fig. 1E). For all mesodermal differentiation experiments, no difference was observed between aMSC and pMSC.

### Adult and pediatric MSC do not exhibit different telomerase activity

**Figure 2:**
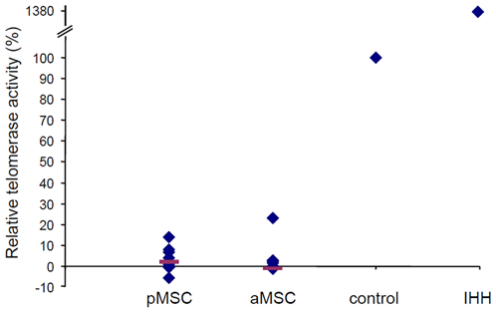
Telomerase activity in adult and pediatric MSC. Telomerase activity of adult and pediatric MSC from 4 different donors and at different passages (1 to 4) was measured and normalized to a provided positive control set as 100 percent. Immortalized human hepatocytes (IHH) were used as quality controls of cell extracts containing telomerase; IHH are immortalized cells transduced with lentivectors coding for telomerase [44]. As compared to control, telomerase activity was low and not significantly different between aMSC and pMSC (p>0.4).

Telomerase activity of MSC in culture remains controversial [16], [17], [21]. We analyzed telomerase activity of aMSC and pMSC in order to determine whether differences in expansion capacities could be related to different activity of telomerase (Fig. 2). Telomerase activity of MSC was measured and normalized to a positive control provided by the assay. IHH, cells transduced with lentivectors coding for telomerase, were used as a positive control for quality of cell extracts. These cells displayed almost fourteen times more telomerase activity than the provided positive control. As shown in figure 2, telomerase activity in MSC extracts was low compared to positive control and not significantly different between aMSC and pMSC (p>0.4).

### Pediatric MSC co-cultured with Huh-7 cells express more frequently albumin and alpha 1 anti-trypsin compared to adult MSC

**Figure 3:**
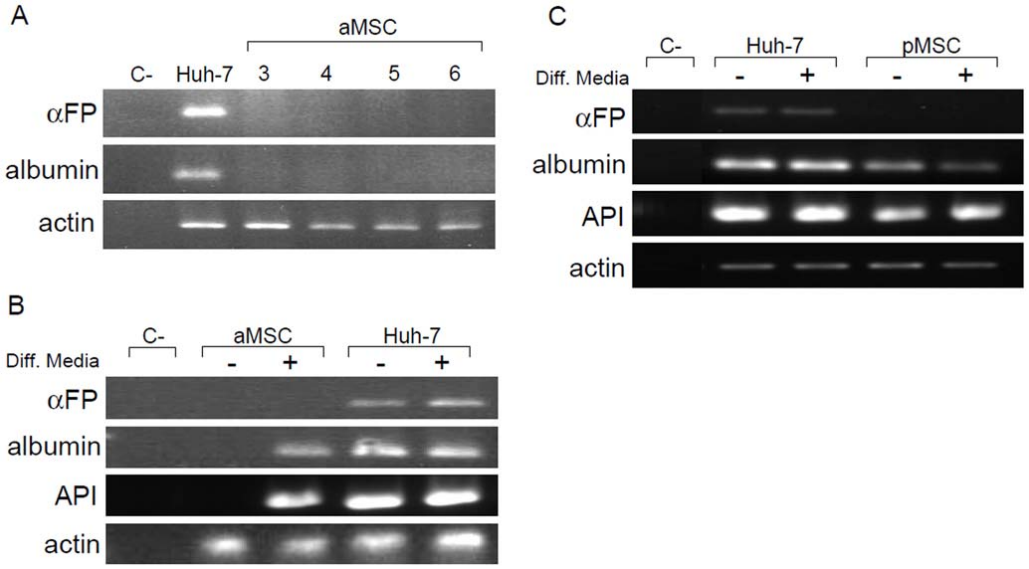
Induction of hepatocyte specific genes in adult and pediatric MSC after co-culture with Huh-7 cells. A) aMSC were cultured for 4 weeks in hepatogenic differentiation medium containing HGF, FGF4 and Oncostatin M. RT-PCR analysis of αFP and albumin expression shows no induction of hepatocyte specific gene expression. C-, negative control, PCR without polymerase. Huh-7, positive control for αFP and albumin expression. Lane 3, 4, 5 and 6: aMSC from different donors. B) Adult MSC were co-cultured with Huh-7 cells in a transwell system, with or without hepatogenic differentiation medium (Diff. Media+/−). C-, negative control. RT-PCR analysis was done on total RNA extracts from aMSC co-cultured with Huh-7 cells for αFP, albumin and API. Huh-7 cells from transwell of same experience were used as a positive control for αFP and albumin. In 2 of 10 independent experiments, we observed in MSC co-cultured with Huh-7 cells in hepatogenic differentiation medium. The result represents one of the two positives results obtained. C) PMSC were co-cultured under same conditions as aMSC. C-, negative control. In 5 of 8 independent experiments, we observed αFP, albumin and API expression in MSC, independently of the presence of hepatogenic differentiation medium. The result represents one of the 5 positives results obtained. API: alpha1 anti-trypsin; αFP: alpha fetoprotein; Huh-7: human hepatoma cell line.

In order to induce hepatocyte differentiation, we cultured aMSC and pMSC at high density on Matrigel coated wells for 24 h and added hepatogenic differentiation medium as previously described [22] containing HGF, fibroblast growth factor 4 (FGF4) and oncostatin M, for 4 weeks. This medium failed to induce alpha fetoprotein (αFP) or albumin expression in both aMSC (Fig. 3A) and pMSC (data not shown). We then co-cultured aMSC or pMSC with Huh-7 cells in hepatogenic differentiation medium in a transwell system preventing direct cell-cell contacts between different cell types. In these conditions, aMSC expressed albumin and alpha 1 anti-trypsin (API) in 2 of 10 independent experiments, and this exclusively in conditions in which hepatogenic differentiation medium were present (Fig. 3B). However, aMSC expressing albumin did not express αFP. By applying identical conditions to pMSC, we observed albumin and API expression in 5 out of 8 independent experiments. Here, in 2 out 5 positive experiments, addition of hepatogenic differentiation medium was not necessary and co-culture with Huh-7 cells was sufficient to induce albumin expression (Fig. 3C). Epithelial markers for hepatoblasts, hepatocytes and cholangiocytes, like cytokeratin 18 (CK18) and cytokeratin 19 (CK19), were expressed in all conditions, in presence or absence of growth factors (data not shown). However, we did not detect albumin at the protein level (data not shown).

### Alpha smooth muscle actin is present in MSC cultured in hepatogenic differentiation medium and in Huh-7 cells conditioned medium

**Figure 4:**
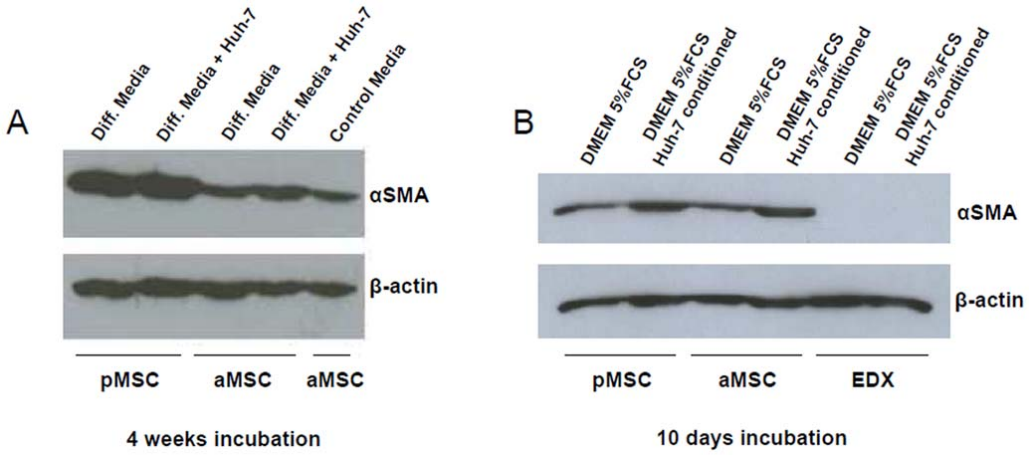
Alpha smooth muscle actin expression in adult and pediatric MSC cultured in various conditions. A) Cell extracts from aMSC and pMSC cultured with or without Huh-7 cells in hepatogenic differentiation medium during 4 weeks were analyzed for their expression of αSMA and compared to aMSC cultured in control condition with low FCS concentration (IMDM 2% FCS) by Western blotting. B) Adult MSC and pMSC and EDX cells where incubated with medium (DMEM 5% FCS) conditioned by Huh-7 cells during 7 days. A and B show representative results of multiple blots. αSMA: alpha smooth muscle actin; aMSC: adult multipotent mesenchymal stromal cells; pMSC: pediatric multipotent mesenchymal stromal cells; EDX: human foreskin fibroblasts.

A recent study indicated that bone marrow cells contribute significantly to hepatic stellate cells and myofibroblasts differentiation in a murine model of liver cirrhosis [23]. We therefore analyzed whether MSC express genes specific to myofibroblasts when cultured in hepatogenic differentiation medium alone or co-cultured with Huh-7 cells in hepatogenic differentiation medium for four weeks. αSMA was expressed strongly in pMSC and to a lesser extent in aMSC when cultured in hepatogenic differentiation medium as well as co-cultured with Huh-7 cells in hepatogenic differentiation medium (Fig. 4A). Adult MSC also express αSMA in control media containing low percentages of FCS to avoid overgrowing during the 4 weeks of culture (Fig. 4A). We then compared aMSC and pMSC with foreskin fibroblasts (EDX) for their ability to express αSMA (Fig. 4B). After culture in Huh-7 cells conditioned medium we observed an up-regulation of αSMA in aMSC and pMSC already at 10 d (Fig. 4B). This was not observed in EDX cells.

### After intrahepatic injection, adult MSC and pediatric MSC engraft but do not differentiate into hepatocytes

**Figure 5:**
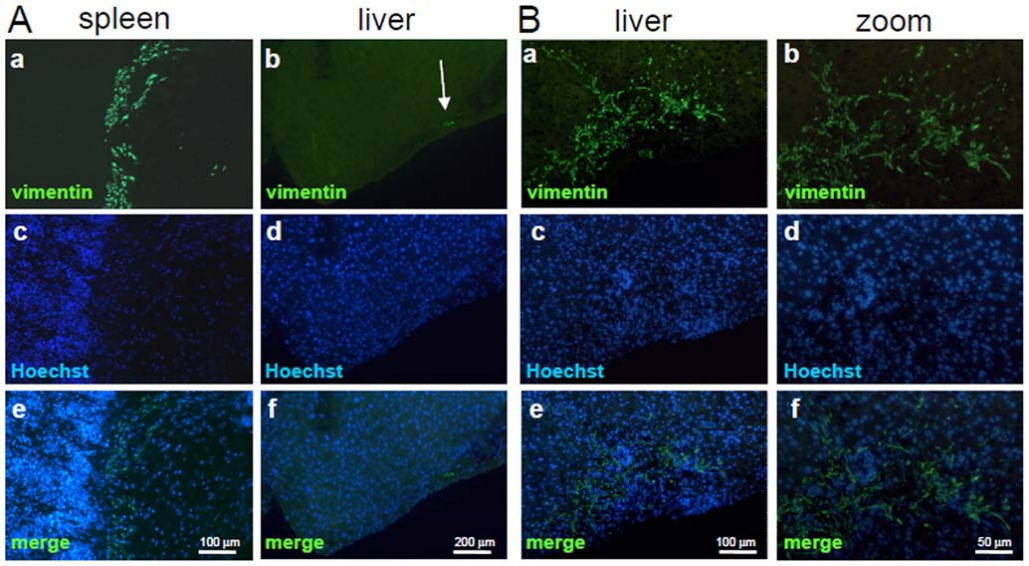
Liver engraftment of MSC in a mouse model of liver injury, after intrasplenic or intra-hepatic transplantation. A) When injected into the spleen of NOD/SCID mice, human adult and pediatric MSC engrafted and survived for 8 weeks in the spleen (a). Only few cells migrated to the liver with maximum 3 cells per high-power field observed (arrow, b). Anti-human vimentin Ab (panel a,b), Hoechst (panel c,d), overlay (lower panel e,f). B) When injected into liver parenchyma, MSC engrafted and high numbers of cells were detected (a,b). However, they retained their spindle shape morphology (b). Anti-human vimentin Ab (panel a,b), Hoechst (panel c,d), overlay (panel e,f). Higher magnification (b,d,f).

 To investigate the engraftment capacity of aMSC and pMSC and their ability to participate in liver regeneration, we injected 0.5 to 1×10^6^ untreated aMSC and pMSC directly into the spleen after 30% or 70% hepatectomy of non-obese-diabetic/severe combined immunodeficient (NOD/SCID) mice (Table 1). By immunofluorescence staining on spleen sections, using an antibody recognizing exclusively human vimentin and not mouse vimentin as verified by western blotting on mouse 3T3 cells (data not shown), we demonstrated that aMSC and pMSC survived in the spleen up to 8 weeks after transplantation (Fig. 5A). Cells expressing vimentin remained fibroblast-like. During the first week after intrasplenic injection, few MSC were detected within the liver (Fig. 5A).

**Table 1:** Summary of in vivo experiments.

Type of cells	% of hepatectomy	Retrorsine	Intrasplenic (S) or intrahepatic (H) injection	Nb of mice	Engraftment spleen	Engraftment liver		Differentiation into hepatocytes
						rate	dist.	
aMSC	30	n	S	23	y	0	0	n
aMSC	30	y	S	2	y	0	0	n
pMSC	30	n	S	25	y	0	0	n
pMSC	70	y	S	11	y	0	0	n
pMSC	70	y	H	8	na	8/8	6:loc. 2:diff.	n
aMSC	70	y	H	8	na	8/8	5:loc. 3:diff.	n

aMSC: adult multipotent mesenchymal stromal cells; pMSC: pediatric multipotent mesenchymal stromal cells; y: yes; n: no; na: not applicable; dist.: distribution;

loc.: localized; diff.: diffuse.

**Figure 6:**
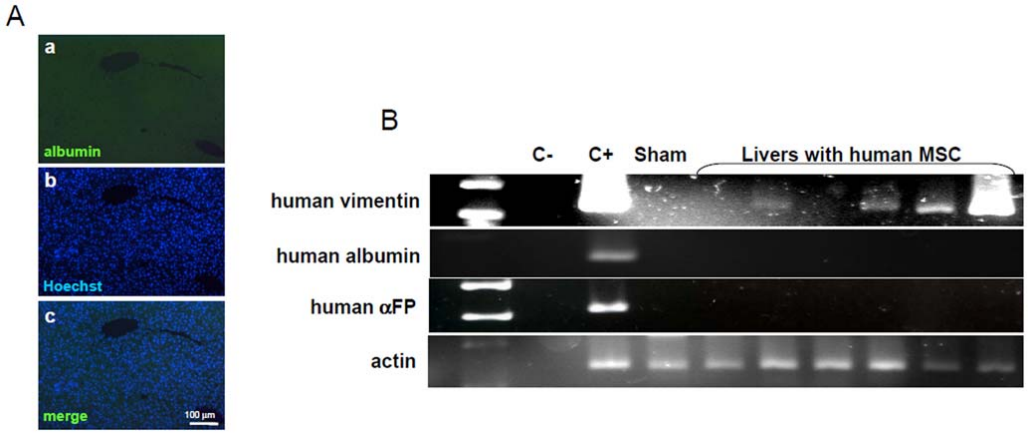
Human albumin is not expressed in mouse liver engrafted with pediatric MSC and adult MSC. A) Staining with anti-human albumin Ab did not detect any albumin positive cells within mouse liver parenchyma (a,b,c). Scale bars indicate magnification. B) Total RNA was extracted from sham injected or transplanted mouse liver. Expression of human vimentin, human αFP and human albumin was analyzed by RT-PCR in several liver tissues after intra-hepatic transplantation with human MSC. Sham: sham injected mouse liver. C+: positive control for human vimentin was RT-PCR on MSC extract. Positive control for αFP and albumin was RT-PCR on human hepatocytes extract. C-: negative control without polymerase. Vimentin was expressed in liver tissue demonstrating engraftment of MSC. Human αFP and albumin were never detected. Each figure shows one representative result of several independent experiments (see table 1).

In order to analyze effects of liver parenchyma on MSC, we injected aMSC and pMSC directly into liver parenchyma. Immunohistochemistry studies showed that pMSC were still present after 8 weeks (Fig. 5B). Extend of engraftment varied from a restricted localization near the border of the liver capsule to a diffuse engraftment in the whole liver lobe where cell injection had been performed (see Table 1: Summary of in vivo experiments). Using an anti-human albumin Ab, we analyzed liver sections at 3 levels. We did not detect human albumin within the liver at any stage after transplantation (Fig. 6A). In accordance to this, RT-PCR on liver samples showed that neither CK18, cytochrome P450 (CYP3A4) (data not shown), αFP, nor albumin but vimentin was expressed, demonstrating that MSC are present but differentiation into hepatocytes did not occur (Fig. 6B). The outcome was identical when hepatectomy was preceded by retrorsine treatment, a treatment blocking endogenous hepatocyte proliferation [24].

### Engrafted MSC in liver express alpha smooth muscle actin and their localization merges with collagen deposition

**Figure 7:**
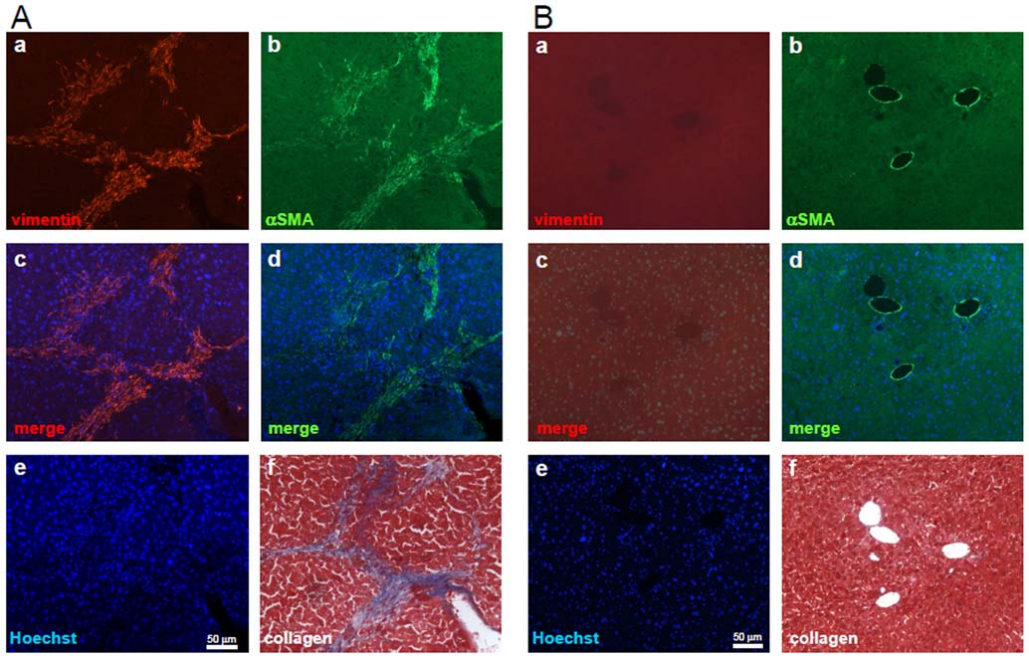
Engrafted MSC express alpha smooth muscle actin and merge with collagen deposition in mouse liver. Eight weeks after injection, sections from MSC injected (A) or sham injected (B) livers were stained with an Ab specific against human vimentin (panels a) or an Ab against αSMA (panels b) or with Hoechst (panels e). MSC retained their spindle like morphology and formed band-like structures similar to fibrotic livers. Overlay images of Hoechst and vimentin (panels c) or Hoechst and αSMA (panels d) are shown. Serial sections were stained with Masson to visualize collagen deposition (blue staining). Comparing collagen stained by Masson (blue) with staining of vimentin and αSMA demonstrate similar localization (Aa, Ab, Af).

During fibrosis, myofibroblasts expressing αSMA appear within the liver. Recently, it has been shown that these cells can be of bone marrow origin [25]. Therefore we investigated whether transplanted MSC could differentiate in myofibroblasts. Staining for αSMA on sections of transplanted mouse liver showed that MSC express αSMA (Fig. 7A). Antibody against αSMA is not human specific and stains smooth muscle cells around blood vessels of mouse livers as shown on liver sections of sham injected mice (Fig. 7B, b and d). Histochemistry using Masson's trichrome on serial sections showed that collagen deposition merges with vimentin- and αSMA-expressing MSC (Fig. 7A, a, b and f).

## Discussion

 MSC are currently tested clinically to treat various bone diseases [26], [27] or graft versus host disease [20]. MSC are also investigated experimentally as cell-based therapies for liver failure. As age might influence plasticity of MSC, we investigated the potential of aMSC and pMSC to engraft and participate in liver regeneration. We first observed that both, aMSC and pMSC can be expanded extensively, up to 18 and 24 PD, respectively. We confirmed previous reports showing that pMSC achieved higher PD and that both cell types reach senescence [28]. This difference in proliferation could not be explained by a difference in telomerase activity. This is in accordance with two recent publications, analyzing proliferation and telomerase activity in MSC [16], [17]. Age-related reduction of colony-forming units as well as increased levels of molecules implicated in cellular aging such as reactive oxygen species and nitric oxide have been observed in MSC [29] and might influence their proliferation capacity.

Qualitative analysis demonstrated that differentiation into adipocytes, osteoblasts and chondrocytes was possible for both MSC populations. Among surface antigens tested, pMSC and aMSC differed solely for expression of CD36. CD36, a scavenger receptor class B1 [30] was not expressed by pMSC. Recent studies showed that CD36 mediates cellular uptake of long chain fatty acids and their intestinal absorption in mice [31]. As both aMSC and pMSC differentiate into adipocytes, CD36 seems not to be important for the *in vitro* differentiation of MSC into adipocytes. The importance of this receptor in MSC biology has not been investigated so far.

In our experiments, induction of hepatocyte-specific genes in MSC failed by using previously reported culture conditions [14], [8], [22]. In contrast, co-culture with Huh-7 cells induced albumin expression in aMSC and pMSC. Pediatric MSC appeared to be more prone to hepatocyte differentiation as co-culture with Huh-7 cells without hepatogenic differentiation medium was sufficient to induce albumin expression. Contamination of underlying MSC with Huh-7 cells from the upper compartment (transwell) was excluded by performing experiments in an inverted cell-setting. We suggest that soluble factors secreted by Huh-7 cells are responsible for induction of hepatogenic gene expression. However, the fact that we did not detect albumin at protein levels suggest that there might only be a small population of MSC differentiating towards hepatocytes.

Other studies using subpopulations isolated from MSC obtained from pediatric donors demonstrated that expression of hepatic genes can be induced [22]. Hepatocyte differentiation was observed *in vitro* in MSC derived from umbilical cord blood [32] or umbilical cord matrix [14]. Campard and colleagues reported that a small proportion of umbilical cord-derived MSC expressed albumin, as shown by flow cytometry. Hepatocyte differentiation has also been observed in human adipose-tissue derived-MSC [33]. There is growing evidence that MSC exhibit potential to express hepatogenic lineage markers but development of significant amounts of functional hepatocytes has still to be achieved.

When cultured in hepatogenic differentiation medium alone or co-cultured with Huh-7 cells in differentiation medium, aMSC and more importantly pMSC expressed αSMA at protein level. Therefore, we suggest that in such conditions the majority of cells develop into myofibroblasts. Incubation of MSC with Huh-7 cells-conditioned medium containing 5% FCS showed that factors secreted by Huh-7 cells induce αSMA expression. Compared to forskin fibroblasts, MSC displayed a higher fibrogenic potential in cell culture. Whether the increased expression of alpha smooth muscle actin is related to the tumorgenic nature of Huh-7 cells has to be determined.

When injected into spleen, after partial hepatectomy, few MSC migrated into the liver and completely disappeared within 7 days as described by others [34]. Other studies achieved low level of engraftment and differentiation of MSC into hepatocytes after intravenous injection or transplantation into portal vein or spleen [35], [36], [33]. It appears that limited transmigration through the endothelial barrier hampers MSC to enter liver parenchyma. In order to expose MSC directly to liver tissue, we injected cells into the remaining 30% of liver and observed long-term survival. However, differentiation of MSC into hepatocytes did not occur as well. Differentiation of human MSC into hepatocyte-like cells after direct injection into liver parenchyma has been described in a rat model of chronic liver injury [37]. Sato and colleagues identified human albumin and αFP-positive clusters in CCl4-treated and immunosuppressed rats. Using an allogeneic rat model, Popp and colleagues reported that MSC do not stably engraft into retrorsine-treated and partially hepatectomized mice [34]. The discrepancy of survival and engraftment of MSC in liver might be related to the immunocompetent model used [34] as clearing of MSC might be under the control of the immune-system [38].

In our study using immunodeficient recipients, we observed long-term engraftment of MSC in liver parenchyma. Histology showed that cells integrated randomly into the tissue without specific distribution. MSC expressed αSMA, a marker for myofibroblasts [39] and vascular smooth muscle cells [40]. In transplanted liver, MSC localization merged with collagen deposition. Injured liver secretes large amount of growth factors and cytokines, i.e. tumor necrosis factor alpha (TNFα), interleukin 6 (IL6) and later transforming growth factor beta 1 (TGFβ1) [41]. Therefore, increased levels of TGFβ may induce migration of MSC through up regulation of molecules such as CD44 [42] and differentiation of MSC into myofibroblast [43].

Fibrogenic potential of adult bone marrow-derived MSC was described in a mouse model of chronic liver injury [36]. Expression of αSMA has also been observed in umbilical cord-derived MSC *in vitro* but their fibrogenic effect had not been confirmed *in vivo* [14]. Despite beneficial effects of MSC on liver fibrosis [13], [12], [11], our data showed that bone-marrow-derived MSC from adult and pediatric donors, transplanted into regenerating liver, displayed fibrogenic activity, indicating a potential harmful effect on liver parenchyma.

In conclusion, *in vitro* albumin expression was induced frequently in MSC derived from pediatric donors but could not be detected at protein level. Further, under such conditions MSC showed increased fibrogenic potential. *In vivo*, both aMSC and pMSC implanted in injured and regenerating liver parenchyma were not able to differentiate into hepatocytes. In the liver parenchyma, MSC remained mesenchymal, expressed αSMA and their localization merged with collagen deposition. These results indicate that adult as well as pediatric MSC were able to develop into fibrogenic tissue in our mice model. Analyzing further the fibrogenic potential of MSC is of strong interest since MSC are currently considered for cell therapy in human.

## Materials and Methods

### Human bone marrow derived multipotent mesenchymal stromal cells isolation and culture

 Human adult and pediatric bone marrow cells were collected from femoral heads, condyles, or bone resections of orthopedic adult and pediatric patients, after written consent from informed patients. This research project was accepted by local Ethical Committees of the Departments of Surgery and Pediatrics, University Hospital Geneva, Switzerland (protocols 01-172/chir01-015 and 04-228/matped04-008). Cells were isolated from bone fragments and cultured as previously described. Medium consisted in Iscove's modified Dulbecco's Medium (IMDM) (Cambrex, Verviers, Belgium), 10% fetal calf serum (FCS) (Gibco-Invitrogen, Basel, Switzerland), 100 IU/ml Penicillin, 100 µg/ml Streptomycin (P–S) (Gibco-Invitrogen), Dithiothreitol (DTT, Sigma, St-Louis, USA) and 10 ng/ml Platelet Derived Growth Factor BB (PDGF-BB, PeproTech EC Ltd, London, UK). Cells were expanded as previously described [5], [7] and used for experiments between passages 3 to 6.

### Culture of human foreskin fibroblasts, human hepatoma cells, mouse 3T3 cells, immortalized human hepatocytes

 Human foreskin fibroblasts (EDX) (a gift from DFB Bioscience) and human hepatoma cells (Huh-7) cells (a gift from Benoît Gauthier, University of Geneva, Switzerland) were maintained in expansion medium consisting of Dulbecco's Modified Eagle Medium (DMEM) (Low Glucose, Gibco-Invitrogen) with 5% FCS for EDX cells and 10% FCS for Huh-7 cells respectively, with P–S at 37°C and 5% CO_2_. Huh-7 cell-conditioned medium (DMEM 5% FCS) was collected every 3 d, filtered and stored at −20°C until use. Immortalized human hepatocytes (IHH) were cultured in medium as described [44]. Mouse 3T3 cells were cultured in DMEM high glucose supplemented with 10% FCS and P–S.

### Antibodies

 Following antibodies (Ab) were used for fluorescent activated cell sorting (FACS) analysis and immunohistochemistry: mouse anti-CD11b (Hycult biotechnology, Uden, the Netherlands), mouse anti-CD31 (Dako, Baar, Switzerland), phycoerythrin (PE)-conjugated mouse anti-CD34, Fluorescein isothiocyanate (FITC)-conjugated mouse anti-CD36, PE-mouse anti-CD44, PE-mouse anti-CD54, mouse anti-CD90, mouse anti-CD106, mouse isotype control (all from Becton Dickinson, Basel, Switzerland), PE-mouse anti-CD45 (R&D systems, Abingdon, UK), FITC-mouse anti-CD105 (Serotec, Oxford, UK), mouse anti-HLA-ABC (Chemicon Australia, Victoria, Australia), mouse anti-vimentin (Dako, Baar, Switzerland), mouse anti-human serum albumin, mouse anti-beta cytoplasmic actin (both from Sigma, Buchs, Switzerland), rabbit anti-human collagen type II (Mono-San, Uden, The Netherlands) and mouse anti-human alpha smooth muscle actin Ab [45]. Secondary Ab were FITC-rat anti-mouse IgG1, PE-rat anti-mouse IgG1 (both from Becton Dickinson), horseradish peroxidase-conjugated goat anti-mouse IgG (Jackson Immunoresearch Laboratories, West Grove, PA), biotinylated mouse anti-rabbit (Dako) and Alexa Fluor 488 (green) goat-anti mouse (Molecular Probes Inc, Eugene, OR).

### Fluorescence Activated Cell Sorting (FACS)

MSC were detached using 0.25% trypsin-EDTA (Sigma, Buchs, Switzerland) for about 30 s and washed twice. Cells were stained with antibodies (Ab) against CD11b, CD31, CD34, CD36, CD44, CD45, CD54, CD90, CD105, CD106, HLA ABC, and mouse isotype control, at saturating concentrations. Cells were washed with phosphate buffered saline (PBS) containing 0.1% bovine serum albumin (BSA) and 0.1% azide (all from Sigma, Buchs, Switzerland) and those labeled with unconjugated Ab were secondary labeled with FITC or PE secondary rat anti-mouse Ab, washed and analyzed using a FACScan flow cytometry system (Becton Dickinson Immunocytometry Systems, Basel, Switzerland). Mouse isotype standard immunoglobulin and secondary Ab alone were used as negative controls. Data were analyzed using Win MDI program (Scripps, La Jolla, Ca, USA).

### Mesodermal differentiation

Adipogenic differentiation: Adult MSC and pMSC were trypsinized and seeded at high density (20′000–25′000 cells/cm^2^). MSC were cultured at 37°C in presence of 5% CO_2_ for 3 weeks on adherent Petri dishes (Falcon, Becton Dickinson, Basel, Switzerland) in adipogenic differentiation media composed of IMDM, 10% rabbit serum (Sigma, Buchs, Switzerland), 0.5 mM 3-Isobutyl-1-methylxanthin (IBMX), 1 µM hydrocortisone, 0.1 mM indomethacin (all from Sigma, Buchs, Switzerland) and P–S. Media were changed every 3 d. Cells were fixed with cold 10% formalin for 1 h, then washed twice with water and stained with Oil-red-O solution (Sigma, Buchs, Switzerland) for 2 h at room temperature (RT), to reveal triglyceride droplets in the cytoplasm. Cells were washed twice, coverslipped and observed under an optical microscope (Zeiss Axiophot1, Carl Zeiss AG, Feldbach, Switzerland).

Osteogenic differentiation: Adult MSC and pMSC were trypsinized and seeded at high density (20′000–25′000 cells/cm^2^). Then, cells were cultured at 37°C with 5% CO_2_ up to 3 weeks on adherent Petri dishes in osteogenic differentiation media based on IMDM, dexamethasone 0.1 µM, β-glycerolphosphate 10 mM, ascorbic acid 200 µM (all from Sigma, Buchs, Switzerland), and P–S. Media was changed every 3 d. Cells were fixed in 10% formalin for 1 h and were either stained with von Kossa staining to detect calcium deposition or stained using alkaline phosphatase activity test. Briefly, for von Kossa staining, after fixation cells were rinsed with distilled water and incubated with a 5% Silver Nitrate solution under strong light for 5 min at RT. After washing with distilled water, coloration was fixed with a 1% sodium thiosulfate solution for 5 min, washed again and nuclei were stained with Nuclear Red for 15 min at RT. Cells were washed twice and observed under an optical microscope (Zeiss Axiophot1). For alkaline phosphatase staining, fixed cells were washed twice with PBS and once with Tris/NaCl (50 mM/150 mM) pH 10 buffer. Cells were then incubated with a mix of 5 vol of a solution of Sodium alpha-naphtyl phosphate (170 µg/ml) and 1 vol of Fast Red TR solution (1 mg/ml) (both from Sigma) for 30 min at 4°C. After washing with PBS, nuclei were stained with Hemalun for 1–2 min at RT and then cells were washed with water, and observed under an optical microscope (Zeiss Axiophot1).

 Chondrogenic differentiation: Adult MSC and pMSC were trypsinized and 300′000 cells were centrifuged at 200×g for 5 min. The pellet was rinsed with PBS and then cultured in a Falcon tube for 3 weeks in a chondrogenic differentiation media based on DMEM – high glucose (25 mmol/l of glucose), 0.1 µM dexamethasone, 50 µg/ml ascorbic acid, 10 mg/l insulin, 5.5 mg/l transferrin, 5 µg/l selenium (ITS)-premix, 40 µg/ml L-proline (all from Sigma, Buchs, Switzerland a), 10 ng/ml TGFβ1 or TGFβ3 (both from PeproTech EC Ltd, London, UK), P–S at 37°C in presence of 5% CO_2_, and media were changed every 3 d. Pellets were fixed with 10% cold formalin for at least 24 h. The pellets were dehydrated, embedded in paraffin. Chondrogenic differentiation was then assessed by immunohistochemistry (see paragraph Immunohistochemistry). 5 µm sections were stained with Masson-Goldner trichrome and Alcian blue to reveal collagen fibrils and glycosaminoglycan, respectively. Slides were mounted and observed under an optical microscope (Zeiss Axiophot 1).

### Hepatic differentiation

Two different protocols were used to induce hepatic differentiation. First, aMSC and pMSC were seeded on 1% Matrigel (BD Biosciences, Allschwil, Switzerland) at a concentration of 20′000–25′000 cells/cm^2^, and attached for 24 h in expansion medium. After 24 h, MSC were cultured in hepatogenic differentiation medium: IMDM, HGF 50 ng/ml, FGF4 50 ng/ml, Oncostatin M 50 ng/ml (all from PeproTech EC, London, England), P–S, DTT or in control medium: IMDM, 2% FCS, P–S, DTT and 10 ng/ml PDGF-BB, at 37°C in presence of 5% CO_2_ for 4 weeks.

Second, aMSC and pMSC were co-cultured with Huh-7 cells in hepatogenic differentiation medium for four weeks. 200′000 Huh-7 cells were seeded in a transwell (0.4 µm mesh, Costar, Baar, Switzerland), and transferred in a 6 well plate (Falcon) in which aMSC or pMSC were previously plated on 1% Matrigel at a concentration of 20′000–25′000 cells/cm^2^. Co-Culture in hepatogenic medium was compared to the co-culture of MSC and Huh-7 cells in control medium (media as above) at 37°C in presence of 5% CO_2_.

### Reverse-Transcription Polymerase Chain Reaction (RT-PCR)

MSC were lysed after 4 weeks of hepatic differentiation. RNA was extracted and RT-PCR was performed as previously described [5]. Expression of αFP, albumin, CK18, CK19 and API was analyzed using specific primers (Table 2). B-cytoplasmic actin expression was used as quality control for reverse transcription.

**Table 2:** PCR primer sets.

Name	Forward	Reverse	°T (°C)	Cycles	Product Size	Genebank reference
αFP	5′-tgcagccaaagtgaagagggaaga-3′	5′-catagcgagcagcccaaagaagaa-3′	59.6	35	217	NM-001134
Albumin	5′-tgctgatgacaggg-3′	5′-gatgagatgcctgctgacttgcctt-3′	60	35	151	NM-000477
API	5′-agaccctttgaagtcaaggacaccg-3′	5′-ccattgctgaagaccttagtgatgc-3′	58	35	359	NM-000295.4
β actin	5′-cgtgggccgccctaggcaccag-3′	5′-ttggccttagggttcagggggg-3′	60	30	243	NM-001101.3
CK18	5′-ctctgggttgaccgtggaggt-3′	5′-tggtgctctcctcaatctgctg-3′	55	35	149	NM-199187
CK19	5′-atggccgagcagaaccggaa-3′	5′-ccatgagccgctggtactcc-3′	58	35	327	NM-002276
Vimentin	5′-gagaactttgccgttgaagc-3′	5′-cgtgatgctgagaagtttcg-3′	51	35	342	NM-003380
CYP3A4	5′-tctcatcccagacttggccat-3′	5′-gaagacagaataacattctt-3′	55	32	300	NM-017460.3

### Telomerase activity

To measure telomerase activity in aMSC and pMSC, we used TeloTAGGG Telomerase PCR ELISA plus (Roche Diagnostics, Mannheim, Germany), according to manufacturer's instruction. Briefly, 200′000 cells of different donors of aMSC and pMSC, and also 200′000 cells of IHH expressing telomerase at high levels as positive controls, were lysed and supernatants were harvested. Cell extracts were then either heat inactivated (negative controls) or used without heat inactivation. Reaction mixture and internal standard were added to each cell extract and transferred to a thermal cycler. Elongation and amplification were performed as described by manufacturer's protocol. Telomerase activity was then quantified by the TRAP reaction, using an ELISA kit with a microplate containing precoated wells, to detect amplification products and thus telomerase activity. Absorbance of samples was measured at 450 nm and activity was expressed as relative telomerase activity, compared to a provided positive control. Relative telomerase activity was calculated by the following formula: [((AbsS−AbsS0)/AbsS,IS)/((Abs+ctrl−Abs+ctrl0)/Abs+ctrl,IS)]*100, AbsS = sample absorbance, AbsS0 = heat inactivated sample absorbance, IS = internal standard, +ctrl = given positive control.

### Protein electrophoresis and immunoblot analysis

After culture of MSC in hepatogenic conditions or in Huh-7 conditioned medium, cells were scraped and thoroughly lysed in sample buffer (62.5 mM Tris-HCl pH 6.8, 2% sodium dodecyl sulfate (SDS), 10% glycerol, 50 mM DTT, 0.01% Bromophenol Blue). Total cell lysates were run on 7.5% SDS minigels [46] (Bio-Rad Laboratories, Glattbrugg, Switzerland) and electroblotted onto PVDF membranes (Millipore, Zug, Switzerland). PVDF membranes were incubated with mouse anti-αSMA Ab and mouse anti beta cytoplasmic actin Ab diluted in Tris-buffered saline (TBS) containing 5% milk, overnight at 4°C. After three washes with TBS, a second incubation was performed with horseradish peroxidase-conjugated affinity-purified goat anti-mouse IgG at a dilution of 1∶6′000 in TBS, containing 5% milk. Peroxidase activity was developed using the ECL western blotting system (Amersham, Rahn AG, Zürich, Switzerland), according to the manufacturer's instructions and blots were scanned (Arcus II; Agfa, Mortsel, Belgium).

### Animals

NOD/SCID mice (Centre Medical Universitaire, Geneva, Switzerland), 8 to 10 weeks old and 20 to 25 g of body weight were used for experiments. Animals were maintained in specific pathogen free housing facilities and experimental protocols were approved by the ethical committee of the Geneva University Medical School and by Geneva veterinary authorities. Mice had ad libitum access to food and water.

### Retrorsine treatment and partial hepatectomy in NOD/SCID mice

In some experiments, mice were treated twice at days −28 and −14 before transplantation with 30 µg/g of body weight of retrorsine (Sigma), an inhibitor of endogenous liver regeneration [34]. Under general anesthesia, a median laparotomy was performed and the left lateral liver lobe (30% hepatectomy) or left lateral, left upper and right anterior lobes (68% hepatectomy) were ligated and removed as previously described by Greene and Puder [47].

### Transplantation of human MSC into partially hepatectomized NOD/SCID mice

 Mice underwent a second laparotomy under general anesthesia, 48 h post-hepatectomy. For intrasplenic injections, undifferentiated MSC suspended in 50–100 µl Hanks' Buffered Saline Solution (HBSS) were injected with a 25 G butterfly catheter. For intrahepatic injections, MSC suspended in 50–100 µl HBSS were injected into two sites of the remaining right lower lobe. Sham animals were operated as transplanted animals and 100 µl of HBSS was injected either into the spleen or the hepatic parenchyma.

### Organ retrieval and fixation

After various time points (from 5 min to 8 weeks), mice were euthanized by exsanguination under general anesthesia and organs were retrieved. Liver and spleen were dissected and fixed in 10% formalin. Organs were dehydrated and embedded in paraffin. Five µm-thick tissue sections were performed at 3 different levels on liver and spleen.

### Immunohistochemistry and histochemistry

All paraffin sections were dewaxed, and rehydrated using xylene and successive ethanol baths.

For detection of mesenchymal cells, hepatic and fibrogenic markers, mouse anti-vimentin (1∶1000), mouse anti-human albumin (1∶250), mouse anti-αSMA (1∶500), were used respectively. Before incubation with the specific Ab, tissue sections were treated with sodium citrate 10 mM at pH 6.0 heated for 10 min at 95°C. For slides incubated with anti-albumin Ab, an additional treatment with image iTTM FX signal enhancer (Molecular Probes), to block non specific antigen was performed. Tissue sections were incubated overnight at 4°C with primary Ab (anti-human vimentin Ab and anti-αSMA Ab) diluted in PBS containing 0.1% BSA, anti-human albumin was diluted in PBS containing 5% FCS). Alexa Fluor (green) 488 goat-anti mouse secondary Ab (1∶1000 in PBS containing 5% goat serum) were incubated for 30 min at RT. Hoechst staining (1∶2000) was performed on certain sections.

For the assessment of chondrogenic differentiation, sections of pellets were stained against collagen type II. Sections were treated with hyaluronidase and chondroitinase (both from Sigma) for 15 min at 37°C. After blocking for 1 h with PBS containing 0.5% BSA, pellets were incubated with rabbit anti-human collagen type 2 Ab (1∶20), overnight, at 4°C. Primary Ab binding was revealed with biotinylated mouse anti-rabbit Ab (1∶250) for 30 min at RT and then with StreptABcomplex and Alkaline phosphatase (Dako, Glostrup, Denmark) for 30 min at RT, according to manufacturer's instruction. Incubation with Fast Red for 5–10 min revealed the antigen. Nuclei were colored by Hemalun.

For histochemistry using Masson's trichrome 48, 49, Slides were then incubated for 1 h at 60°C in Bouin's solution as mordant. Slides were washed in tap water for 5 min. Nuclei were stained with hematoxylin. Slides were washed with tap water for 5 min. Cytoplasm was stained with Biebrich scarlet for 5 min. Slides were rinsed in distilled water, incubated in phosphomolybdic acid for 10 min, transferred into Aniline blue for 5 min to stain collagen and rinsed in distilled water. They were then rinsed in 2% acetic acid for 1 min, rapidly dehydrated in ethanol 95% and 100%, followed by xylol.

Stained slides were coverslipped and tissue sections were observed using an Axiophot microscope (Carl Zeiss AG, Feldbach, Switzerland) and images were acquired with an Axiocam color camera (Zeiss).

### Statistics

Results were analyzed by Student's. P-values of p≤0.05 were considered statistically significant.
