# Correction: Fibrogenic Potential of Human Multipotent Mesenchymal Stromal Cells in Injured Liver

**DOI:** 10.1371/annotation/0c224e4f-d48d-4c12-adfa-f2afd7b9a62f

**Published:** 2009-10-09

**Authors:** Reto M. Baertschiger, Véronique Serre-Beinier, Philippe Morel, Domenico Bosco, Marion Peyrou, Sophie Clément, Antonino Sgroi, André Kaelin, Leo H. Buhler, Carmen Gonelle-Gispert

There was an error in affiliation 3 for author Sophie Clément. Affiliation 3 should be: Division of Clinical Pathology, Medical School of Geneva, Geneva, Switzerland.

